# Protocol for the external validation of machine learning models for risk prediction in early-onset preeclampsia: a multicenter prospective cohort study

**DOI:** 10.1186/s12884-026-09424-3

**Published:** 2026-06-11

**Authors:** Paula Domínguez-del Olmo, Iñigo Melchor, Esther Cánovas, Pablo García-Manau, Marc Josep Cahuana, María Pérez-Marqués, Elena Satorres, Fernando Bugatto, Cecilia Villalaín, Eva Robisco, José L Ayala, Alberto Galindo, Ignacio Herraiz

**Affiliations:** 1 Department of Computer Architecture and Automation, Faculty of Informatics, Complutense University of Madrid, Madrid, Spain; 2 Spanish Network in Maternal, Neonatal, Child and Developmental Health Research (RICORS-SAMID), RD24/0013/0015, Instituto de Salud Carlos III, Madrid, Spain; 3 Department of Obstetrics and Gynecology, Hospital Universitario Cruces, Instituto de Investigación Sanitaria BioBizkaia, Barakaldo, Bizkaia Spain; 4 Department of Obstetrics and Gynecology, Hospital Universitario Virgen de La Arrixaca, IMIB/Arrixaca, Murcia, Spain; 5 Department of Obstetrics and Gynecology, Hospital de la Santa Creu i Sant Pau, Barcelona, Spain; 6 BCNatal - Barcelona Center for Maternal-Fetal and Neonatal Medicine (Hospital Clínic y Hospital Sant Joan de Deu), Institut Clínic de Ginecología, Obstetrícia I Neonatología, Fetal i+D Fetal Medicine Research Center, Institut d’Investigacions Biomèdiques August Pi I Sunyer (IDIBAPS), Universidad de Barcelona, Barcelona, Spain; 7 Department of Obstetrics and Gynecology, Hospital Clínico Universitario Lozano Blesa, Zaragoza, Spain; 8 Department of Obstetrics and Gynecology, Hospital Universitari i Politècnic La Fe, Valencia, Spain; 9 Department of Child and Mother Health and Radiology, School of Medicine, University of Cádiz and Division of Maternal and Fetal Medicine, Department of Obstetrics and Gynecology Puerta del Mar University Hospital, Cádiz, Spain; 10 Biomedical Research and Innovation Institute of Cádiz (INiBICA), Cádiz, Spain; 11 Department of Obstetrics and Gynecology, Hospital Universitario 12 de Octubre, Madrid, Spain; 12 Instituto de Investigación Hospital 12 de Octubre (imas12), Madrid, Spain; 13 Facultad de Medicina, Universidad Complutense de Madrid (UCM), Madrid, Spain

**Keywords:** Preeclampsia, Machine learning, Validation, Prediction, Multicenter, Clinical decision support system

## Abstract

**Background:**

Early-onset preeclampsia (eoPE), defined as preeclampsia occurring before 34 weeks of gestation, remains one of the leading causes of severe maternal morbidity, iatrogenic prematurity, and maternal mortality worldwide. Its heterogeneous and rapidly evolving clinical course makes the prediction of adverse outcomes particularly challenging. Although machine learning (ML)–based models have shown promise in stratifying eoPE risk and supporting clinical decision-making, robust prospective external validation is still lacking. The present study aims to address this gap through a multicenter validation of three ML models previously developed by our group to: (1) predict maternal complications, (2) estimate the likelihood of delivery within ≤ 7 days of diagnosis, and (3) identify the risk of hemolysis, elevated liver enzymes, and low platelets (HELLP) syndrome or placental abruption.

**Methods:**

This prospective multicenter cohort study (2024–2027) recruits pregnant women diagnosed with eoPE after informed consent. Clinical, analytical, and ultrasound data, including all variables used in the models, are recorded from early pregnancy to delivery. The first 100 cases will undergo quantitative external validation of three ML models and a semi-quantitative evaluation by clinicians, after which these cases will be used to retrain the models. The updated models will then be prospectively applied to the next 100 participants. Model predictions and physician´s assessment of the clinical utility of such predictions are generated through a secure web application accessible only after delivery.

**Discussion:**

This study addresses a critical gap in the prospective validation of ML-based tools for eoPE, emphasizing methodological rigor, data quality, and external validation in a multicenter setting. By integrating quantitative performance assessment with clinician-centered evaluation, the project seeks to ensure both statistical robustness and real-world clinical relevance. If successfully validated, these models could support more individualized and timely decision-making in eoPE management.

**Supplementary Information:**

The online version contains supplementary material available at 10.1186/s12884-026-09424-3.

## Background

Preeclampsia (PE) is a hypertensive disorder of pregnancy characterized by the de novo onset of hypertension after 20 weeks of gestation, accompanied by proteinuria or evidence of end-organ dysfunction or fetal growth restriction (FGR) [1]. Complications such as eclampsia, placental abruption and hemolysis, elevated liver enzymes, and low platelets (HELLP) syndrome leading to life-threatening conditions like liver hematoma and disseminated intravascular coagulation, make PE one of the leading causes of maternal morbidity and mortality worldwide. The incidence of PE is estimated at 2–5%, and it accounts for approximately 50,000 maternal deaths each year, 99% of which occur in low- and middle-income countries [2, 3]. Optimizing antenatal care to prevent disease progression is therefore essential to mitigate the severity and adverse outcomes associated with the disorder [4]. However, delivery remains the only definitive treatment, making early forms of PE the leading cause of iatrogenic prematurity [5, 6]. Early-onset preeclampsia (eoPE), defined as PE occurring before 34 + 0 weeks of gestation, accounts for about 20% of all cases and is associated with a tenfold increase in maternal mortality and a fourfold increase in severe maternal morbidity [7].

In eoPE, an excess of antiangiogenic factors—particularly soluble fms-like tyrosine kinase-1 (sFlt-1)—is released into the maternal circulation, reducing the bioavailability of proangiogenic factors such as placental growth factor (PlGF). The resulting angiogenic imbalance induces systemic endothelial dysfunction, which underlies the multiorgan involvement characteristic of the disease [8]. An elevated sFlt-1/PlGF ratio has been consistently associated with the onset and severity of eoPE [9] and shows an inverse correlation with the time to delivery [10]. These findings highlight the potential role of angiogenic biomarkers for improving clinical risk stratification.

The clinical management of eoPE remains complex, as clinicians must balance the maternal risks of disease progression against the potential neonatal benefits of pregnancy prolongation. In the absence of complications, most international guidelines recommend delivery at 34 weeks’ gestation to optimize neonatal outcomes [11]. However, prolonging pregnancy to reach this threshold carries significant maternal risks [12]. Observational data indicate that approximately one in three women managed expectantly experiences a major maternal complication requiring intensive care, including HELLP syndrome (12%), placental abruption (11%), pulmonary edema (3%), or renal failure (4%) [13]. The strategy of continuing pregnancy until 34 weeks is based primarily on two clinical trials conducted in the 1990s [14, 15], and a subsequent systematic review incorporating more recent studies concluded that further evidence is needed to establish its safety for the mother [12]. Even under optimal conditions and with careful case selection, the median duration of pregnancy prolongation achieved through expectant management is around seven days (standard deviation: 2–18 days) [14]. While this short gain may be crucial for neonatal outcomes in extremely preterm stages, its benefit becomes less justifiable beyond 30 weeks, when neonatal survival rates in tertiary centers reach 97% at 30–32 weeks (90% without severe morbidity) and 99% at 32–34 weeks (98% without severe morbidity) [16–18].

Another clinical difficulty lies in the administration of corticosteroids for fetal lung maturation. A complete course administered within the week prior to birth significantly reduces neonatal morbidity and mortality, with an adjusted odds ratio of 1.46 (95% CI: 1.20–1.77) compared to administration more than a week before delivery [19]. However, repeated courses appear to be counterproductive, particularly in the long term [20]. Current recommendations therefore favor a single course administered within seven days of delivery [21], a goal that is difficult to achieve in eoPE because of the unpredictable rate of disease progression [22].

Together, these clinical uncertainties underscore the need for accurate, individualized tools capable of predicting the short-term risk of maternal complications and the likelihood of requiring delivery in the days following diagnosis. Several predictive models have been developed to assist clinicians in estimating the risk of complications in PE, although their applicability to eoPE remains limited [23, 24]. The most popular is the PIERS model. Although initially promising, it has shown some limitations. Validation studies have demonstrated that its performance is highly dependent on the case-mix and healthcare setting [25–28]. Additionally, its predictive horizon is restricted to 48 h, as it relies on variables that are direct indicators of established organ dysfunction (rather than angiogenic markers), thus offering limited usefulness for anticipating disease progression [29].

On the other hand, Machine Learning (ML) offers a promising approach to address this challenge, as it can capture complex, nonlinear interactions among clinical, ultrasound, and laboratory variables (including angiogenic markers) while minimizing human bias in interpretation [23]. Building on our previous work [13], our group recently developed a ML–based ensemble model using a cohort of 234 patients diagnosed with eoPE. Within this framework, women diagnosed with eoPE are initially assessed using the main model, “Risk of Adverse Outcomes” (R), which estimates the probability of developing any of six major maternal complications: pulmonary edema, renal failure, refractory hypertension, neurological events, HELLP syndrome, or placental abruption. For those classified as high risk, two additional predictive models are subsequently applied: the “Time to Delivery” (T) model that predicts the need for delivery within seven days of diagnosis and the “HELLP-Abruption” (HA) model that estimates the risk of these two acute and unexpected adverse outcomes. This ensemble modeling framework is designed to support individualized decision-making regarding timely hospitalization, corticoid administration and delivery [30].

**Fig. 1:**
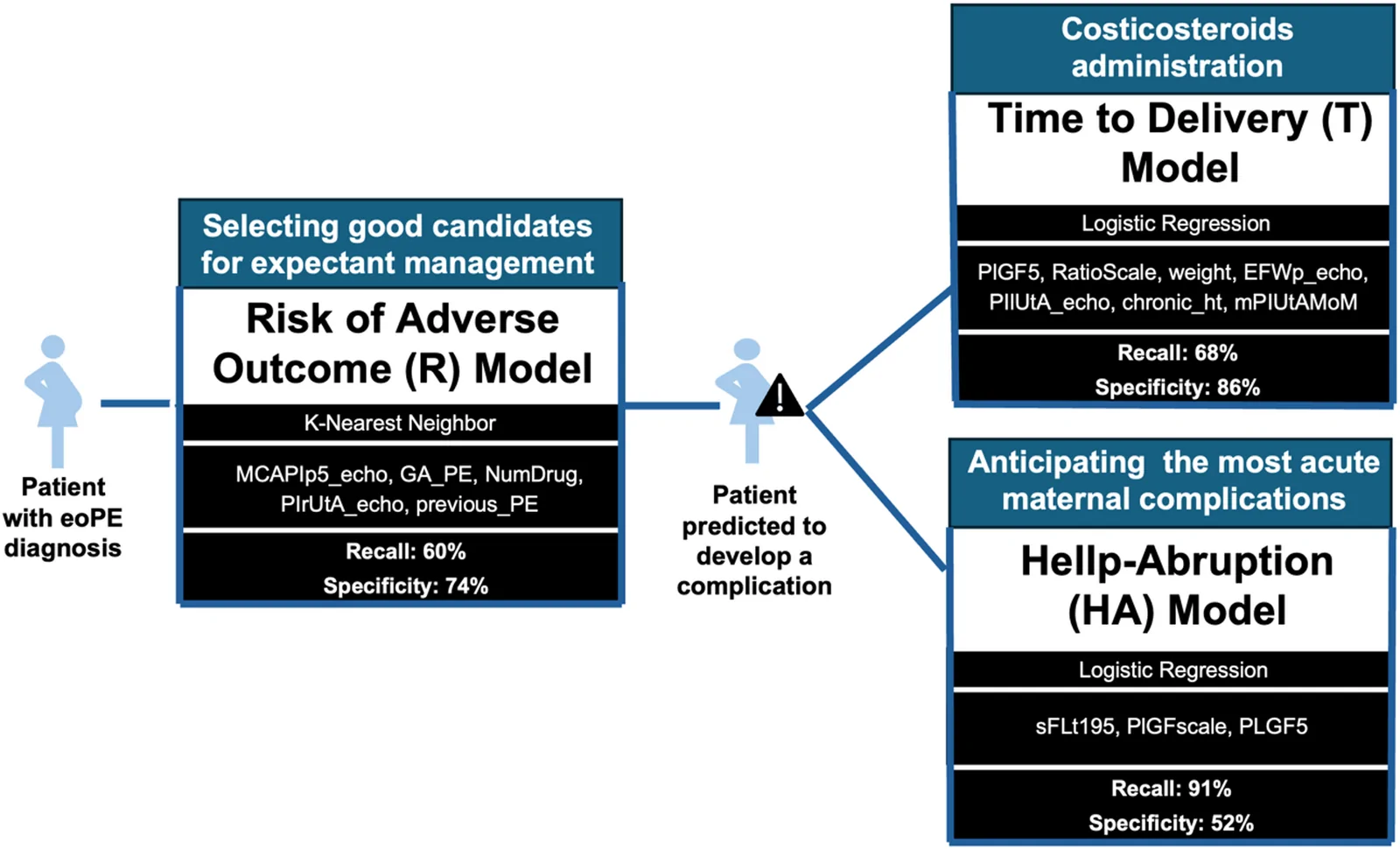
Overview of the machine learning models to be validated. For each model, the classification approach, the input variables used, and the results obtained during the development phase are presented. MCAPIp5_echo: Pulsatility index in the middle cerebral artery < percentile 5; GA_PE: Gestational age at preeclampsia onset; NumDrug: Number of antihypertensive administrated drugs; PIrUtA_echo: Pulsatility index of the right uterine artery; previous_PE: Preeclampsia in previous gestations; PlGF5: Angiogenic marker < percentile 5 for gestational age according to Verlohren et al. [32]; RatioScale: Clinical classification of sFlt-1/PlGF relation (0–38: normal; >38–85: suspicion of preeclampsia; >85–655: consistent with a diagnosis of preeclampsia; >655: high-risk of preeclampsia-related complications); weight: Pre-gestational weight; EFWp_echo: Customized percentile of estimated fetal weight; PIlUtA_echo: Pulsatility index of the left uterine artery; chronic_ht: Maternal chronic hypertension; mPIUtAMoM: Multiples of the median of the mean pulsatility index of the uterine arteries; sFLt195: Antiangiogenic marker > percentile 95 for gestational age according to Verlohren et al. [32]; PlGFscale: Clinical classification of PlGF

The development process required addressing the complexity and heterogeneity of the dataset so a structured feature–selection pipeline was implemented using genetic algorithms (GAs), an evolutionary computation technique capable of efficiently searching high-dimensional feature spaces. Two complementary strategies were applied: a mono-objective GA aimed at maximizing the F1-score of each classifier, and a multi-objective GA (NSGA-II) that simultaneously optimized predictive performance and model parsimony, favouring solutions with fewer variables to enhance clinical feasibility [31]. The three models underwent internal validation and demonstrated promising diagnostic performance (sensitivity and specificity of 70%/74%, 69%/88% and 88%/69%, respectively). The R model (K-Nearest Neighbor) included twenty-five predictive variables, the T one (Logistic Regression) included fourteen, whereas the HA (Logistic Regression) incorporated thirteen. Subsequently, simplified versions of the models were derived from the multi-objective strategy: based on only five, seven and three features, achieving a sensitivity of 60%/68%/92% and a specificity of 74%/86%/52% (Fig. 1) [30]. Although the HA model prioritizes sensitivity over specificity, this trade-off may be clinically acceptable, particularly between 30 + 0 and 33 + 6 weeks of gestation, when the maternal risks of continuing the pregnancy increase while neonatal outcomes improve rapidly with gestational age.

Despite these encouraging findings, external validation in a broader and more heterogeneous population is essential before these models can be considered for clinical implementation. The present study aims to address this need assessing the diagnostic accuracy, calibration, and clinical utility of the models across multiple hospitals participating in the Spanish RICORS-SAMID network.

## Methods

### Study setting

This is a prospective, multicenter, observational cohort study that is conducted at six Spanish sites including Hospital Universitario 12 de Octubre in Madrid, Hospital Sant Joan de Déu and Hospital de la Santa Creu i Sant Pau in Barcelona, Hospital Universitario Cruces in Barakaldo in Bizkaia, Hospital Lozano Blesa in Zaragoza, Hospital Universitario Virgen de La Arrixaca in Murcia and Hospital Universitari i Politècnic La Fe in Valencia. Additionally, Hospital Universitario Puerta del Mar in Cádiz are planned to be incorporated soon.

### Dates of the study

The study is planned to run from 2024 to 2027 and is currently in the recruitment phase. Participant recruitment began on October 1, 2024, and is expected to be completed by the end of 2026. Data will be collected in parallel with patient recruitment, from the beginning of pregnancy until delivery. The data collection period will then be extended for an additional two months to complete follow-up for the last enrolled participants, ensuring that all required information is available for the final analysis. Results are expected by May 2026.

### Objectives

The primary objective of this study is to externally validate the diagnostic performance and clinical usefulness of three ML–based models designed to predict (1) maternal complications, (2) the need for delivery within ≤ 7 days of diagnosis, and (3) the development of HELLP syndrome or placental abruption.

Secondary objectives of this study include:

- Improvement of the prediction models by incorporating data from 100 new eoPE cases into the ML training process.
- Adjustment of the training process to specifically train and optimize the models for the eoPE subgroup with the greatest potential for applicability due to their clinical interest in management: PE diagnosed between 30 + 0 and 33 + 6 weeks of gestation.
- Conduct a semi-quantitative post-hoc assessment of healthcare professionals’ satisfaction with the information provided by the models to adjust the timing of (1) fetal maturation with corticosteroids, (2) hospitalization, and (3) pregnancy termination.
- Development of a web application that integrates the data collection and validation process (both quantitative and semi-quantitative) into a single tool accessible to clinicians.

### Inclusion criteria

Eligible patients are pregnant women meeting all of the following criteria: (1) maternal age ≥ 18 years; (2) singleton pregnancy; (3) diagnosis of PE established according to the criteria of the International Society for the Study of Hypertension in Pregnancy [11]; (4) gestational age at the time of diagnosis between 20 + 0 and 33 + 6 weeks; (5) availability of angiogenic biomarker results (sFlt-1/PlGF ratio) obtained at the time of diagnosis (± 3 days); and (6) provision of written informed consent to participate in the study.

### Exclusion criteria

Patients will be excluded if: (1) do not provide written informed consent to participate in the study; (2) fetal congenital malformations are present, such as severe structural anomalies, genetic disorders, or confirmed congenital infections; (3) delivery occurs at a non-participating center.

### Sample size estimation

We will evaluate 5 candidate predictors for the R model, 7 for the T model, and 3 for the HA model as specified in Fig. 1. Following the recommendation of having at least 10 events per predictive variable and assuming that:

- Approximately 50% of women with eoPE deliver within ≤ 7 days [14], the required sample size to validate the T model is: 7 (predictive features) x 10 (events) / 0,5 (event incidence) = 140 eoPE cases
- Approximately 40% of women with eoPE experience any PE-related complication, and in 20% these complications include placental abruption or HELLP syndrome [30]. According to this, the required sample size to validate the R model is: 5 (predictive features) x 10 (events) / 0,4 (event incidence) = 125 eoPE cases, and to validate the HA model is: 3 (predictive features) x 10 (events) / 0,2 (event incidence) = 150 eoPE cases

This estimation assumes independence among predictor variables. However, ML techniques can identify dependencies between variables when present, which could potentially reduce the required sample size. Nonetheless, we have chosen to maintain the estimate of 200 cases, as it is the most conservative assumption and aligns with the sample size used in our previous work during model development, providing greater confidence.

### Study variables

#### Dependent variables

Below are the outcomes assessed in each model:

- R Model: presence of at least one of the following adverse maternal events (yes/no):
  - ◦ Pulmonary edema confirmed radiologically and associated with oxygen saturation <95%.
  - ◦ Acute kidney injury (creatinine ≥1.6 mg/dL or an increase ≥1.0 mg/dL from baseline) or oliguria (<500 mL/24 h).
  - ◦ Refractory hypertension: severe and uncontrolled elevation of blood pressure for >12 hours despite the use of at least three antihypertensive medications.
  - ◦ Neurological events: hemorrhagic stroke, coma (Glasgow Coma Scale <13), posterior reversible encephalopathy syndrome, or cortical visual loss including retinal detachment.
  - ◦ HELLP syndrome: hemolysis (bilirubin ≥1.2 mg/dL, LDH ≥600 IU, or diagnosis by peripheral smear), elevated transaminases (AST ≥70 IU/L), and platelet count <100,000/mm³. Cases meeting two of the three criteria (incomplete HELLP) will also be included.
  - ◦ Placental abruption: partial or complete separation of the placenta from its normal uterine implantation site before completion of the second stage of labor. Diagnosis requires evidence of an organized retroplacental hematoma involving at least 20% of the implantation area.
- T model: interval between the diagnosis of eoPE and delivery ≤7 days (yes/no)
- HA model: presence of HELLP syndrome or abruptio placentae (yes/no)

#### Independent variables

The models draw on maternal demographic information and clinical data obtained at the time of eoPE diagnosis, including laboratory parameters, angiogenic biomarkers (sFlt-1/PlGF), and ultrasound findings. Below, the predictor variables used in each specific model are described in detail:

- R model features: pulsatility index in the middle cerebral artery < percentile 5, gestational age at PE onset, number of antihypertensive administrated drugs, pulsatility index of the right uterine artery, previous PE
- T model features: PlGF < percentile 5, sFlt-1/PlGF clinical classification, weight, personalized percentile of estimated fetal weight, pulsatility index of the left uterine artery, chronic hypertension and multiples of the median of the mean pulsatility index of the uterine arteries
- HA model features: sFLt1 > percentile 95, PlGF clinical classification, PlGF < percentile 5

The models do not incorporate information related to the care center or socioeconomic status. They also do not include data on perinatal care; however, the study does collect the main perinatal outcomes for descriptive purposes. The complete set of variables collected for this study is detailed in Additional file 1.

### Study regimen

**Fig. 2:**
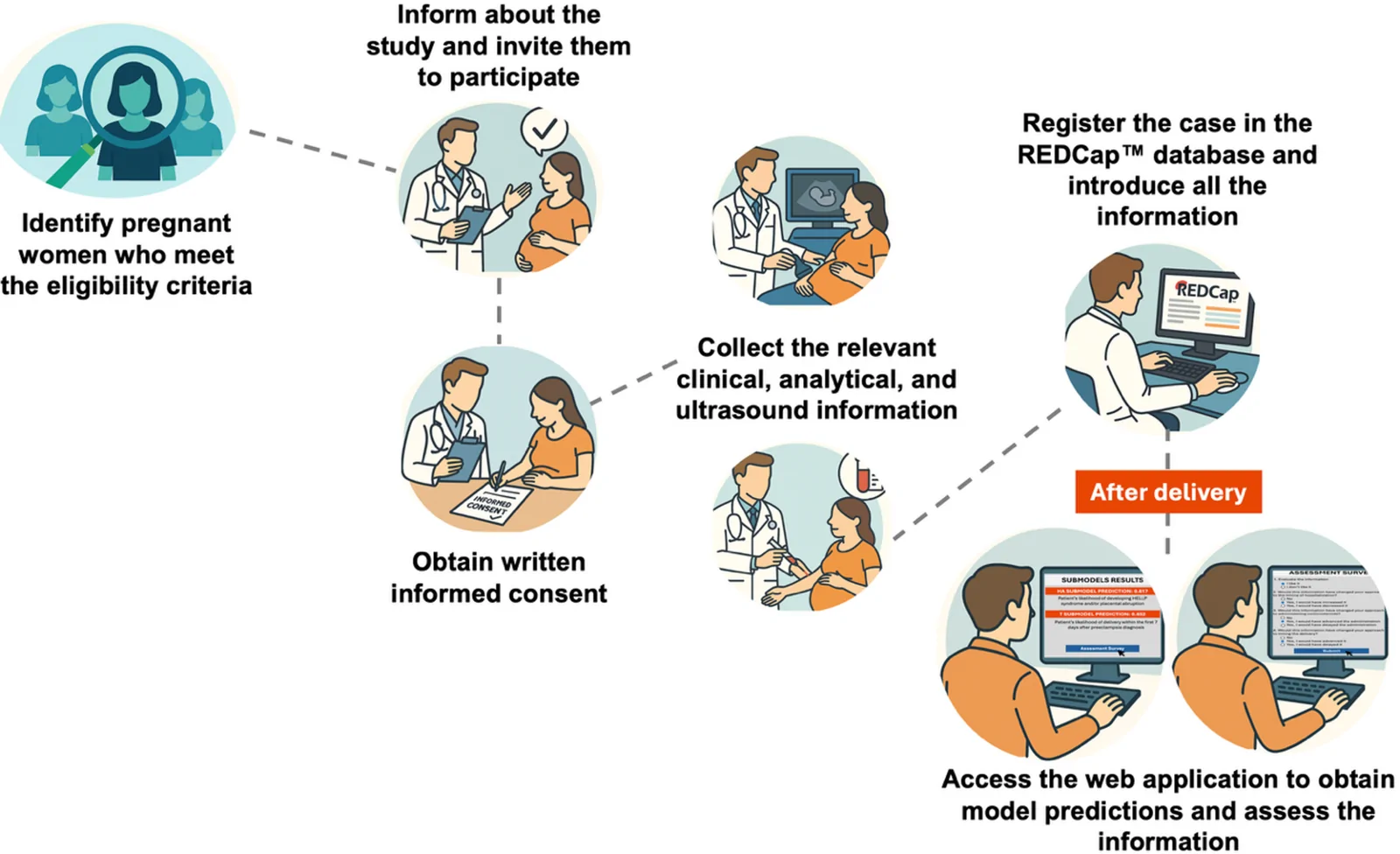
Study regimen including recruitment, data collection, and models validation

The study will follow the steps outlined in Fig. 2. First, pregnant women who meet the eligibility criteria will be identified at each participating center. They will receive both verbal and written information explaining the purpose and procedures of the study and will be invited to participate. After consent, the investigator will register the case in the REDCap™ database [33], hosted by the Research Institute of Hospital 12 de Octubre (i + 12), by selecting the appropriate center in the “Data Access Group,” which automatically generates a unique study identifier. All relevant clinical, analytical, angiogenic, and ultrasound data, from early pregnancy through delivery, will be collected and entered into the corresponding REDCap™ sections. Only once delivery has occurred will the clinician access the web application to obtain the model predictions and complete the semi-quantitative assessment, following the procedure detailed in the “Web Application” section.

### Data collection

Data collection constitutes a critical phase of the study, as the quality and completeness of the information recorded will directly determine the robustness and validity of the subsequent external validation analyses. Study data are entered and managed is performed through a dedicated database developed for this purpose on the REDCapTM platform. It allows participating centers to create and complete electronic case report forms in real time and supports data import and export in multiple formats, including Microsoft Excel, PDF, SAS, Stata, R, and SPSS.

The designated clinician at each participating center will be responsible for recording all patient data in REDCap™, covering the period from the start of pregnancy until delivery. Each participant will be assigned a unique identification code that decouples clinical data from personally identifiable information. The linking file containing the correspondence between study codes and personal identifiers will be stored securely and accessible only to a limited number of authorized persons. Thus, the data complies with the Personal Data Protection and Guarantee of Digital Rights and Regulation (EU) 2016/679 of the European Parliament and of the Council of April 27, 2016 on Data Protection (GDPR).

In this sense, the first and last sections of the database are particularly important and are described in detail in Additional file 1. In the first section (“Patient ID”), the clinician must enter the patient’s medical record number (MRN), which is immediately encrypted to ensure full anonymization. The clinician must also confirm that the inclusion and exclusion criteria are met and record the date on which informed consent was signed.

In the final section (“Semi-quantitative assessment”), a link to the web application is provided. The appearance of the application interface is shown in Additional file 2. Once the data used by the models and the delivery information have been entered into the database, the clinician can access the application to obtain the model outputs and complete the semi-quantitative assessment. Both the model outputs and the clinicians’ responses will be automatically stored in this section under the corresponding patient’s record. This retrospective appraisal minimizes optimism bias and allows the evaluation to reflect the clinical value of the predictions rather than responses influenced by the models themselves.

### Models’ validation strategy

An analysis will be conducted after the inclusion of the first 100 participants to perform an initial external validation of the predictive models. This validation will include both quantitative and semi-quantitative assessments:

- The quantitative evaluation will focus on the diagnostic performance of the predicted risks, including the estimation of sensitivity, specificity, positive and negative predictive values, discrimination using the area under the ROC curve, calibration using the Hosmer–Lemeshow test, and clinical utility assessed through decision curve analysis.
- The semi-quantitative evaluation will consider clinicians’ perceptions of the information provided by the models. After reviewing the model’s information, they will be asked to answer four questions to assess the perceived usefulness and potential clinical impact of the predictions: (1) overall appreciation of the information (“I like it / I don’t like it”); (2) whether this information would have influenced their decision regarding corticosteroid administration (“No / Yes, I would have administered them earlier / Yes, I would have administered them later”); (3) whether it would have modified their management of hospitalization between diagnosis and delivery (“No / Yes, I would have extend the duration of hospitalization / Yes, I would have reduced it”); and (4) whether it would have changed their decision about the timing of delivery (“No / Yes, I would have indicated delivery earlier / Yes, I would have delayed delivery”).

Following this first phase, the ML training methodology will be replicated incorporating the information from the initial 100 cases, and the updated models will then be applied prospectively to the next 100 participants. This “re-training” methodology begins with a preprocessing stage, which includes missing-value imputation using MissForest, outlier removal, standardization of numerical variables with Standard Scaler, and encoding of categorical variables using OneHot and Label encoding [34]. Subsequently, model training is carried out using genetic algorithms within a multi-objective framework. These algorithms are non-deterministic, can be adaptive, and often benefit from parallel computation, enabling them to search for high-quality approximate solutions [35].

After completing recruitment of 200 patients, a final validation will be performed, formally comparing the discriminative performance of the original and retrained models using the DeLong test for paired ROC curves. Bootstrap resampling will also be considered to derive more robust confidence intervals for key performance metrics, ensuring a stable evaluation of model behavior across samples. In parallel, the semi-quantitative responses from clinicians will be compared between the two phases, providing both an objective and a subjective assessment of model performance and clinical usability.

### Web application tool

A dedicated web application has been developed to integrate the ML models into the study workflow and to facilitate both data entry and risk estimation once delivery has occurred. The application was built in Python 3.9 using Flask, a lightweight Python-based web framework that allows efficient and flexible development. The full pipeline adheres to good ML practice principles, including version control of model artefacts, centralized logging of prediction events and critical errors, and full traceability of data exchanges with REDCap™ through structured API calls. For reproducibility and transparency, the full list of Python packages, libraries, and environment dependencies used to implement the web application is provided in Additional file 3.

**Fig. 3:**
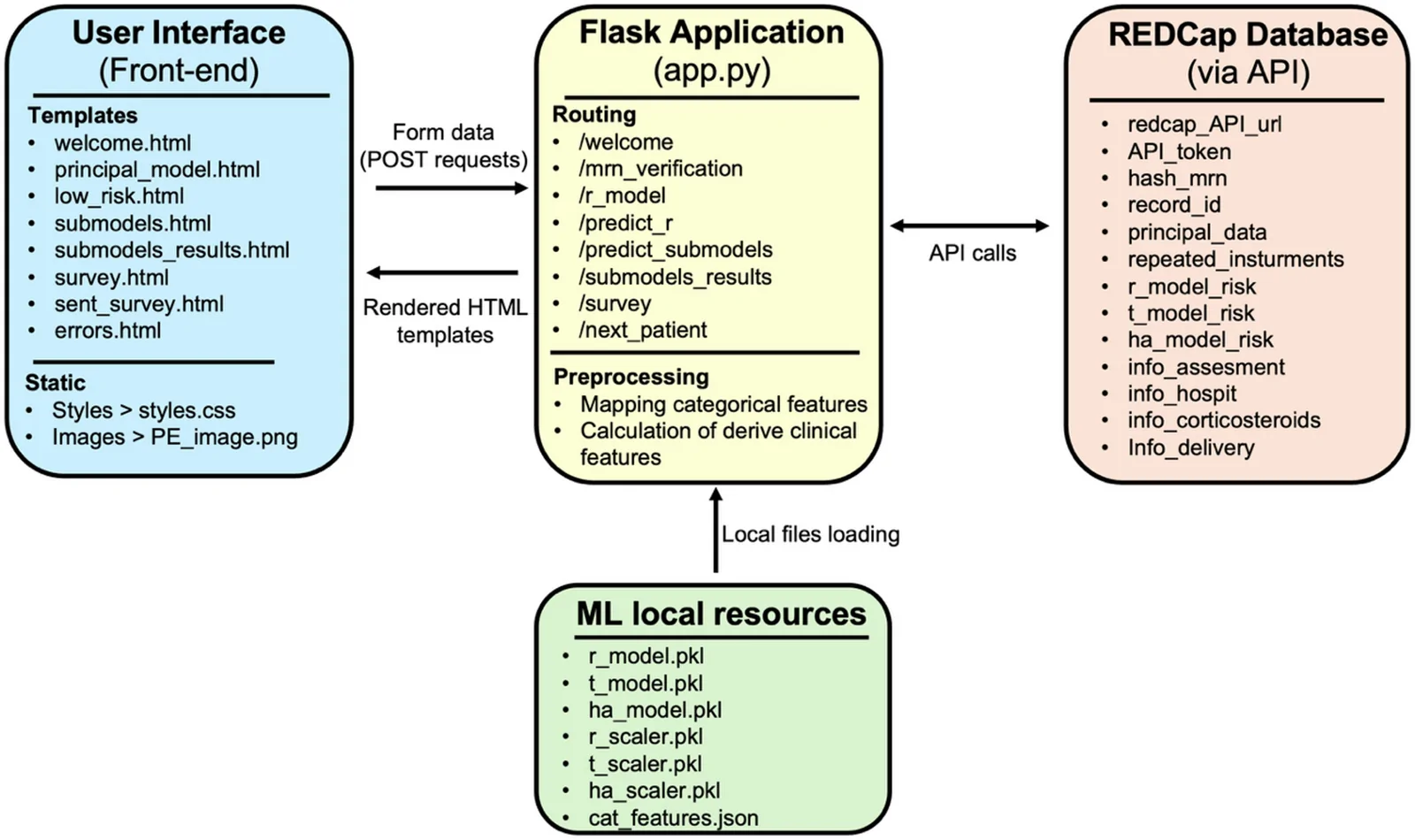
Software architecture of the web-based application tool

As represented in Fig. 3, the system is structured in four layers. The user interface (blue box, Table 1) consists of HTML templates which allow clinicians to enter patient data, view model outputs, and complete the post-hoc survey; and static resources which contains a cascading style sheet (styles.css) that control layout and design, improving project organization and scalability.

**Table 1:** Web application HTML templates and associated functionalities

HTML Template	Function
welcome.html	Displays the initial page where the clinician enters the clinical record number. Initiates the workflow that verifies or creates the REDCap™ record
principal_model.html	Main input form for the R model. Allows manual entry or prepopulation of clinical, laboratory, and ultrasound data retrieved from REDCap™
low_risk.html	Informs the clinician that the predicted risk of maternal complications is low (≤ 0.5), indicating that no additional models (HA or T) are required. Provides navigation to the post-prediction survey
submodels.html	Displayed only when the R model predicts a high risk (> 0.5). Collects the additional variables needed to run the HA and T submodels.
submodels_results.html	Presents the prediction results generated by the HA and T submodels. Provides navigation to the post-prediction survey
survey.html	Form allowing clinicians to assess the usefulness of the model outputs after reviewing the predictions
sent_survey.html	Confirmation page indicating that the clinician’s survey responses were successfully recorded in REDCap™. Provides navigation to the welcome page if the clinician wants to introduce a new patient
errors.html	Displays context-specific error messages (invalid API token, inconsistent REDCap™ records or failed data retrieval), guiding the user back to the start of the workflow

All interactions are handled by the Flask application (yellow box, Table 2), which manages routing, session states, data preprocessing, generation of clinical derived variables, prediction requests to the ML models, and communication with the REDCap™ API.

**Table 2:** Web Application Routing Structure and associated functionalities

Route	Function
/welcome	Provides navigation to the initial page
/mrn_verification	It is triggered when the clinician enters a MRN in the initial form. Its function is to encrypt the MRN, check whether it already exists in REDCap™, and based on that, either load the corresponding data or create a new record, before redirecting the user to /r_model
/r_model	Shows the main form for the R model, prepopulated with REDCap™ data when available. Validates the API token before loading
/predict_r	Processes all variables for the R model: computes derived parameters, normalizes numeric variables, encodes categorical features, stores key variables in session, runs the R model, obtains the prediction, stores the result in session and determines whether submodels are required
/predict_submodels	Retrieves from the session the variables shared with the R model, processes additional variables for the HA and T models, computes derived parameters, runs both models, obtains the predictions, stores results in session, and redirects to /submodels_results
/submodels_results	Provides navigation to the page that displays the predicted risks for HA model T model. Retrieves record_id from session and sends the models probabilities to REDCap™
/survey	Provides navigation to the page that displays the post-prediction survey. Collects clinician feedback, retrieves record_id from session, sends all information to REDCap™, and confirms successful submission
/next_patient	Clears the session (preserving the API token) and resets the workflow for a new patient

The trained models, their associated scalers, and the categorical-variable mapping file are stored locally on the server as serialized resources (green box) and are loaded by Flask at runtime. The REDCap™ database (red box) acts as the system’s secure data repository. It provides patient information used to prepopulate the web forms and receives the outputs of the prediction models as well as the clinicians’ survey responses. Communication between the web application and REDCap™ is performed through its Application Programming Interface (API), using a site-specific API token that enables authenticated access. All data exchange occurs via POST requests sent to the REDCap™ API endpoint, ensuring secure, structured transfer of clinical information throughout the workflow.

### Workflow

Upon accessing the application, the user enters the patient’s MRN on the welcome page. This identifier is encrypted using a one-way hash function before any query is made. The application connects to the REDCap™ database to check whether the encrypted medical record number exists.

- If the encrypted medical record number exists, the corresponding record ID is retrieved and the associated data are automatically loaded into the input forms.
- If it does not exist, a new REDCap™ record is created with the encrypted identifier, and the forms are displayed empty for manual data entry.

After entering the clinical, analytical, and ultrasound variables, the R model provides the predicted probability of maternal complications.

- If the predicted risk exceeds 0.5, the forms for the HA and T submodels are automatically displayed. Once these variables are entered, both predictions are generated, and the clinician proceeds to complete the survey. The model outputs together with the survey responses are then sent to REDCap™.
- If the predicted risk is lower than 0.5, the clinician is redirected directly to the survey, and the probability estimated by the R model along with the survey responses is stored in REDCap™.

Finally, the application allows the user to reset the session and initiate the workflow for a new patient.

### Data handling

Categorical variables are presented as dropdown menus (< select> fields) containing predefined options. A mapping dictionary stored in a JSON file ensures consistent translation between numeric codes from REDCap™ and their corresponding descriptive labels.

Continuous numeric variables are collected through number input fields, with predefined minimum, maximum, and step values to prevent out-of-range inputs. Discrete numeric variables are captured through range sliders, allowing quick selection within a defined interval. All form fields are marked as required to ensure data completeness before submission.

The application can compute derived variables internally. For example, it calculates maternal age from the date of birth and the estimated gestational age at diagnosis from the last menstrual period. Similarly, variables such as mean arterial pressure are automatically derived from systolic and diastolic pressures and stored in the session for reuse across models. This prevents users from re-entering redundant information and ensures internal consistency of the data.

### Server deployment and data security

The application is deployed on a secure institutional server managed by the research team. The server infrastructure defines the domain through which the Flask application is accessed and ensures that all processing occurs within a controlled environment. No data migration or external transfer is performed at any stage; all computations, storage, and exchanges with REDCap™ take place entirely within the protected server ecosystem. The system adheres to institutional policies and European data protection regulations, ensuring encrypted communication, restricted access based on authorized roles, and full traceability of all operations.

## Discussion

The integration of emerging technologies, including ML and biomarkers, into perinatal medicine calls for a reassessment of current strategies for eoPE. Existing approaches remain overly simplistic and are increasingly misaligned with the principles of personalized medicine, which aim to deliver individualized care for patients affected by complex and heterogeneous conditions [36].

Although ML offers substantial opportunities for advancing predictive and personalized medicine, its indiscriminate application entails significant risks. The development of robust and clinically reliable ML-based models critically depends on data quality, adequate sample size estimation, and rigorous validation procedures [37]. These requirements pose particular challenges in the context of rare conditions such as eoPE; nevertheless, they represent challenges that must be addressed. In this regard, our prospective, multicenter study design—aimed at both training and validating predictive models using sufficiently large and high-quality datasets—seeks to promote a cautious, transparent, and methodologically sound implementation of ML-based tools.

The successful validation of these predictive models could serve as a foundation for subsequent research, including randomized clinical trials comparing standard management with strategies informed by individualized, model-based risk assessment at the time of eoPE diagnosis. Ultimately, this may contribute to optimizing clinical decision-making related to timely hospitalization, corticosteroid administration, and delivery.

## Supplementary Information

Additional file 1: Description of the collected study variables.

Additional file 2: Web Application Interface.

Additional file 3: Description of the Python libraries used for the development of the Web Application tool.

## Data Availability

The results generated by this study will be presented at academic conferences and published in international peer-reviewed journals. The datasets generated and/or analyzed during the current study are not publicly available due to ethical and private restrictions but are available from the corresponding author Ignacio Herraiz (ignacio.herraiz@salud.madrid.org) on reasonable request.

## Abbreviations

eoPE: Early—onset preeclampsia
ML: Machine learning
HELLP: Hemolysis, elevated liver enzymes and low platelets
PE: Preeclampsia
FGR: Fetal growth restriction
sFlt-1: Soluble fms—like tyrosine kinase—1
PlGF: Placental growth factor
R: Risk of Adverse Outcomes
T: Time to Delivery
HA: HELLP—Abruption
GA: Genetic algorithm
GDPR: General Data Protection Regulation
MRN: Medical record number
