# Supplementary material for: Protocol for the external validation of machine learning models for risk prediction in early-onset preeclampsia: a multicenter prospective cohort study
Source: BMC Pregnancy Childbirth. 2026 Jun 11;26:951. doi: 10.1186/s12884-026-09424-3 (PMC13508443; doi:10.1186/s12884-026-09424-3)
Supplement: Supplementary file 1 [file 12884_2026_9424_MOESM1_ESM.docx]

Additional file 1: Distribution of variables across REDCap™ data collection sections

| **Patient Identification** | Medical record number  **Inclusion criteria:** Singleton pregnancy, age >/= 18 years old, preeclampsia, gestational age at diagnosis between 20+0 and 33+6, sFlt-1/PlGF available at diagnosis (+/-3 days), signed consent form for participation.  **Exclusion criteria:** congenital malformation, malformation, genetic anomaly, congenital fetal infection.  Date of informed consent signing |
| --- | --- |
| **Baseline Demographics** | Patient’s date of birth, last menstrual period, patient’s age at the beginning of pregnancy, height, pregestational weight, body mass index, smoking habits, ethnicity, method of conception, donor egg in vitro fertilization, number of previous pregnancies, number of previous vaginal deliveries, nulliparous or previous pregnancy 10 or more years ago, number of previous cesarean sections and miscarriages, chronic hypertension, chronic renal disease, pregestational type 2 diabetes, thrombophilia, systemic erythematous lupus, family history of preeclampsia. |
| **Prophylactic and protective measures** | First trimester preeclampsia combined screening, preventive antiplatelet medication (low dose aspirin), anticoagulant medication, corticosteroid prenatal administration, date of last corticosteroid dose, magnesium sulfate administration, legal pregnancy termination, intrauterine death. |
| **Ultrasound data** | Timing of ultrasound, date of ultrasound, gestational age at ultrasound, biparietal diameter, head circumference, abdominal circumference, femur length, estimated fetal weight, right uterine artery pulsatility index, left uterine artery pulsatility index, mean uterine artery pulsatility index, umbilical artery pulsatility index, end-diastolic flow in the umbilical arteries, middle cerebral artery pulsatility Index, cerebroplacental ratio, ductus venosus pulsatility index, ductus venosus flow. |
| **Fetal Growth Restriction (FGR) data** | Date of FGR diagnosis, gestational age at diagnosis, FGR stage at diagnosis and at delivery. |
| **Preeclampsia data** | **At diagnosis:** Date of preeclampsia diagnosis, gestational age at diagnosis, systolic, diastolic and mean blood pressure at diagnosis, method of proteinuria assessment at preeclampsia diagnosis, protein-to-creatinine ratio (mg/dL) in a random urine sample at preeclampsia diagnosis, tendon reflexes at preeclampsia diagnosis, pulse oximetry (O₂ saturation, %) at preeclampsia diagnosis, medication at preeclampia diagnosis.  **During clinical course of preeclampsia:** maximum systolic, diastolic and mean blood pressure, use of antihypertensive medication, preeclampsia complications. |
| **Hematological and biochemical test results** | Timing of the test, date of the test, sFlt-1 level, PlGF level, sFlt-1/PlGF ratio, platform used to measure the angiogenic markers, hemoglobin, platelets, creatinine, uric acid, AST/GOT, ALT/GPT, LDH, albumin, calcium, sodium. |
| **Delivery outcomes** | Indication for pregnancy delivery, date of delivery, gestational age at delivery, Labor induction, Type of delivery, indication for cesarean section, neonatal sex, birth weight, Apgar test score, umbilical cord artery pH. |
| **Neonatal outcomes** | Death before neonatal discharge, admission in neonatal intensive care unit, bronchopulmonary dysplasia, intraventricular hemorrhage grade III or more, periventricular leukomalacia, necrotizing enterocolitis grade 2 or more, prematurity retinopathy grade III or more, neonatal sepsis, neonatal acidosis, hemodynamic instability requiring vasoactive drugs. |
| **Semiquantitative evaluation** | **Predictive model results:** Probability of maternal complication, Probability of HELLP syndrome or placental abruption, Probability of delivery within ≤7 days,  **Semi-quantitative assessment of model information by healthcare professionals:** Assessment of the information provided, Healthcare professionals’ opinion on if this information had been known at diagnosis, would it have changed their approach regarding corticosteroid administration, hospitalization (between preeclampsia diagnosis and delivery), or the timing of delivery. |
