# Supplementary material for: Protocol for the external validation of machine learning models for risk prediction in early-onset preeclampsia: a multicenter prospective cohort study
Source: BMC Pregnancy Childbirth. 2026 Jun 11;26:951. doi: 10.1186/s12884-026-09424-3 (PMC13508443; doi:10.1186/s12884-026-09424-3)
Supplement: Supplementary file 2 [file 12884_2026_9424_MOESM2_ESM.docx]

Additional file 2. Figures showing the web application and REDCap integration.

Figure 1 : Web application home page.

Figure 2: Input data for the R model.

Figure 3: R model prediction and input data for the HA and T submodels.

Figure 4: HA and T submodels predictions

Figure 5: Survey to evaluate the information provided by the models.

Figure 6: Web Application final page

Figure 7: Final REDCap section (“Semi-quantitative Evaluation) before and after using the web application
