# Supplementary material for: Protocol for the external validation of machine learning models for risk prediction in early-onset preeclampsia: a multicenter prospective cohort study
Source: BMC Pregnancy Childbirth. 2026 Jun 11;26:951. doi: 10.1186/s12884-026-09424-3 (PMC13508443; doi:10.1186/s12884-026-09424-3)
Supplement: Supplementary file 3 [file 12884_2026_9424_MOESM3_ESM.docx]

Additional file 3: Software Environment and Python Libraries Used in the Web Application

| **Category** | **Library / Component** | **Function** |
| --- | --- | --- |
| **Web framework** | Flask (render_template, request, redirect, url_for, session) | Routing, session management, page rendering |
| **Model loading and serialization** | joblib | Load ML models, scalers, artefacts |
| **Numerical computing** | numpy | Vector/matrix operations |
| **Statistical functions** | scipy.stats.norm | Normal distribution and percentiles |
| **Mathematical operations** | cmath.exp | Exponential functions |
| **HTTP communication** | requests | POST calls to REDCap™ API |
| **Data handling** | json, pandas | Parse JSON, tabular data processing |
| **Datetime utilities** | datetime | Age calculation, gestational age estimation |
| **Security / Hashing** | base64, hmac, hashlib | SHA-256 hashing of encrypted identifiers |
| **System access** | os, sys | Server-side environment management |
| **Environment variables** | python-dotenv (load_dotenv) | Secure API token and environment config |
